# Editorial: Novel Insights Into the Immunology of Pulmonary Granulomatous Diseases

**DOI:** 10.3389/fimmu.2020.633103

**Published:** 2021-01-05

**Authors:** Mary Jane Thomassen, Marc A. Judson, Barbara P. Barna

**Affiliations:** ^1^Division of Pulmonary, Critical Care and Sleep Medicine, East Carolina University, Greenville, NC, United States; ^2^Division of Pulmonary, Critical Care, Albany Medical College, Albany, NY, United States

**Keywords:** granulomatous disease, sarcoidosis, Th17, matrix metalloproteinase 12, environmental and occupational exposures

Pulmonary granulomatous disease encompasses both infectious and non-infectious disease forms. Presently, the immunopathogenesis of granulomatous inflammation is incompletely understood. This issue of Frontiers in Immunology explores recent insights concerning the clinical and immunological aspects of pulmonary granulomatous disease.

Sarcoidosis is a complex multi-system disease of unknown cause which can be related to a diverse collection of environmental signals. The review by Judson summarizes current knowledge of the immunopathogenesis of sarcoidosis as well as the nature of varied environmental and occupational exposures, which could cause or affect the disease. Many infectious agents have been implicated in the disease, especially mycobacterium tuberculosis, and the review summarizes evidence for sarcoid patient immune responsiveness to such agents.

A different perspective on pulmonary tuberculosis, another granulomatous disease, is provided by Muefong and Sutherland in their review of neutrophil activities in this deadliest of infectious diseases in humans. The authors raise the possibility that neutrophils may play a more central role in tuberculosis pathogenesis than previously thought. They propose that some neutrophil-related inflammatory mediators as potential targets for developing novel tuberculosis therapies.

Hena reviews the effects of race on sarcoidosis epidemiology. The author presents data that show that in the United States, black patients with sarcoidosis experience more severe pulmonary disease, more multiorgan involvement, and an overall worse prognosis. The author proposes that epidemiologic concepts can be used to modulate and even prevent sarcoidosis in black Americans at risk of life-threatening disease phenotypes.

Kraaijvanger et al. address the problem of identifying useful biomarkers for sarcoidosis diagnosis and prognosis. As noted by the authors, sarcoidosis is a heterogeneous disease, making treatment decisions problematic. Exploration of signaling pathways in sarcoidosis such as JAK/STAT and mTOR may result in the discovery of more specific biomarkers that may be relevant to prognosis. The authors stress that there is an unmet need for more specific and more sensitive biomarkers in sarcoidosis care.

Gerke focuses on the deficiencies of the current treatment options for sarcoidosis. Sarcoidosis treatment decisions are problematic because of the marked heterogeneity of the disease. Clinical evidence is sparse because the disease is relatively rare and few clinical trials have been performed. Goals of treatment are to protect organ function and to decrease symptoms, but most clinical sarcoidosis trials have not focused on the impact of the disease on function or quality of life. Gerke emphasizes the lack of knowledge concerning etiology and pathophysiology of the disease and the need to utilize more patient-centered approaches to treatment for exploration of immunosuppression, symptom control, and treatment of co-morbid conditions.

Relevant to the Gerke study, Talreja et al. present original research on alveolar macrophage and blood monocyte gene profiles in response to *in vitro* dexamethasone treatment in sarcoidosis. Effects of glucocorticoids on alveolar macrophages or monocytes have not been well documented. These authors describe profound transcriptosomal changes in lung macrophages relative to cellular metabolism, lysosomal phagosomes and cytoskeleton function. Further studies are proposed to examine proteome profiles in additional cell types relevant to pulmonary sarcoidosis that can correlate with RNA seq data and give a more comprehensive picture of dexamethasone effects within the lung.

Mohan et al.’s study using a multiwall carbon nanotube model of chronic granulomatous disease demonstrated many similarities to sarcoidosis including high expression levels of MMP12 (matrix metalloproteinase). These investigators used MMP12 knock-out mice to investigate the role of MMP12 in granuloma formation and persistence and demonstrated similar acute responses (10 days); however; at day 60, there was a marked reduction in granuloma persistence suggesting that MMP12 is essential for the persistence of granulomas.

Arger et al. observed that T-Bet expression on Th17.0 cells reflected the extent of clinical granuloma burden in sarcoidosis patients. These authors postulated that T-Bet Th17.0 cells represent a transition state leading to interferon-producing Th17.1 cells which are elevated in BAL fluid and mediastinal lymph nodes of sarcoidosis patients with granulomatous inflammation. These studies emphasize the importance of Th17 in sarcoidosis.

Greaves et al. performed a comparative review of adaptive immunity in two pulmonary diseases: pulmonary sarcoidosis and chronic beryllium disease (CBD). The authors note that one of the defining features of both CBD and sarcoidosis is the pulmonary infiltration of activated CD4+ T lymphocytes. Interestingly, sarcoidosis lung also contains a significant population of Th17 cells which are absent in CBD. While beryllium is the antigen that can activate CBD lymphocytes, the antigenic specificity of sarcoidosis lymphocytes is not yet known.

Locke et al. reviewed various *in vitro* and *in vivo* models of sarcoidosis. Each of the current model systems has strengths and weaknesses. The authors suggest that the integrated use of the various preclinical models will accelerate progress toward identifying targets and the testing of new drugs.

We hope that the articles in this issue of Frontiers in Immunology will educate researchers and clinicians concerning the immunology of pulmonary granulomatous disease. Furthermore, we hope that these articles will serve as a stimulus to further exploration of the mechanisms of granuloma formation that will ultimately improve the lives of patients.

## Author Contributions

All authors contributed equally. All authors contributed to the article and approved the submitted version.

## Funding

This study was funded by grants ES025191 to MT and CHHE P30 ES025128.

## Conflict of Interest

The authors declare that the research was conducted in the absence of any commercial or financial relationships that could be construed as a potential conflict of interest.

